# Altered Effective Connectivity of the Primary Motor Cortex in Transient Ischemic Attack

**DOI:** 10.1155/2022/2219993

**Published:** 2022-11-18

**Authors:** Zeqi Hao, Yulin Song, Yuyu Shi, Hongyu Xi, Hongqiang Zhang, Mengqi Zhao, Jiahao Yu, Lina Huang, Huayun Li

**Affiliations:** ^1^School of Teacher Education, Zhejiang Normal University, Jinhua, China; ^2^Key Laboratory of Intelligent Education Technology and Application, Zhejiang Normal University, Jinhua, China; ^3^Department of Neurology, Anshan Changda Hospital, Anshan, China; ^4^Faculty of Western Languages, Heilongjiang University, Harbin, China; ^5^Department of Radiology, Changshu No. 2 People's Hospital, The Affiliated Changshu Hospital of Xuzhou Medical University, Changshu, Jiangsu, China

## Abstract

**Objective:**

This study is aimed at exploring alteration in motor-related effective connectivity in individuals with transient ischemic attack (TIA).

**Methods:**

A total of 48 individuals with TIA and 41 age-matched and sex-matched healthy controls (HCs) were recruited for this study. The participants were scanned using MRI, and their clinical characteristics were collected. To investigate motor-related effective connectivity differences between individuals with TIA and HCs, the bilateral primary motor cortex (M1) was used as the regions of interest (ROIs) to perform a whole-brain Granger causality analysis (GCA). Furthermore, partial correlation was used to evaluate the relationship between GCA values and the clinical characteristics of individuals with TIA.

**Results:**

Compared with HCs, individuals with TIA demonstrated alterations in the effective connectivity between M1 and widely distributed brain regions involved in motor, visual, auditory, and sensory integration. In addition, GCA values were significantly correlated with high- and low-density lipoprotein cholesterols in individuals with TIA.

**Conclusion:**

This study provides important evidence for the alteration of motor-related effective connectivity in TIA, which reflects the abnormal information flow between different brain regions. This could help further elucidate the pathological mechanisms of motor impairment in individuals with TIA and provide a new perspective for future early diagnosis and intervention for TIA.

## 1. Introduction

Transient ischemic attack (TIA) is defined as a brief episode of neurological dysfunction caused by focal cerebral ischemia that does not result in acute cerebral infarction [1, 2]. Individuals with TIA have been clinically observed to be at high risk of suffering a stroke, and 7.5%–17.4% of individuals with TIA have a stroke within 3 months [3–5]. Nevertheless, timely treatment of individuals with TIA could reduce the risk of stroke by 80% in 3 months [6–8], which has received increasing attention in recent years [9, 10]. To improve the diagnosis and treatment, it is essential to better understand the underlying neural mechanisms of TIA [11–14].

Resting-state functional magnetic resonance imaging (rs-fMRI) is a promising tool for investigating neurophysiological mechanisms [15–17] and has been extensively used to examine alterations in spontaneous neural activity in individuals with TIA [18–21]. In rs-fMRI, functional connectivity (FC) is an extensively adopted method for examining the brain network characteristics of TIA and disturbances in connectivity have been revealed in previous studies of TIA [22–24]. However, FC only reflects the correlation of time series between brain regions and ignores the causal effect of the interactions [25–27]. Previous studies have demonstrated that effective connectivity could reveal the causal effect of the interactions between brain areas [28–30], which is how damaged brain areas influence other brain areas in a particular direction [31, 32]. Granger causality analysis (GCA) is one of the commonly used effective connectivity methods that can measure causal effects and information flow of fMRI time series, thus reflecting the directionality of interactions between brain regions [33–38]. In contrast to other effective connectivity methods, such as dynamic causal modeling and structural equation modeling, GCA is relatively data driven and does not require a priori anatomical models for data analysis and statistical inference [39, 40]. The method is therefore particularly suitable for exploratory studies on brain interaction in the context of a limited understanding of the pathological mechanisms of TIA, which could provide further evidence of how the disease affects the brains of individuals.

Motor impairment is a typical transient symptom associated with TIA [6, 41, 42], which mainly includes impaired limb dexterity, limb weakness, limb numbness, gait disturbance, and loss of coordination [43–45]. Previous studies have demonstrated that motor impairment is also a key high-risk clinical characteristic of subsequent stroke [46, 47]. TIA patients with motor impairment are twice as likely to have a stroke as those without motor impairment [48]. The primary motor cortex (M1) plays a critical role in the network responsible for voluntary motor functions [49, 50]. The execution of motor commands is accomplished through neuronal signals generated and sent by M1 [51–53] and is closely associated with motor impairment in TIA [54–56]. Therefore, using bilateral M1 as the regions of interest (ROIs) to explore the alteration of motor-related effective connectivity in TIA could advance our understanding of the pathological mechanisms of TIA.

In the current study, we used the GCA with bilateral M1 as ROIs to examine whether individuals with TIA had altered effective connectivity compared to healthy controls (HCs). Subsequently, we investigated the relationship between alterations of effective connectivity and clinical characteristics. According to our hypothesis, there were alterations in effective connectivity in individuals with TIA, which were related to clinical characteristics.

## 2. Materials and Methods

### 2.1. Participants

A total of 51 participants with suspected TIA were enrolled at Anshan Changda Hospital between April 2015 and June 2016. Experienced clinical neurologists evaluated all participants for neurological symptoms associated with a possible vascular etiology. Individuals with a current or past history of hemorrhage, leukodystrophy, migraine, epilepsy, or psychiatric or neurological disorders were excluded. In addition, we recruited 41 HCs with no history of psychiatric or neurological disorders via community advertising, matching their age and sex to participants with TIA.

The MRI data of three individuals with TIA were excluded from further analysis because of their poor image quality. Finally, 48 individuals with TIA (25 with nonfirst TIA and 4 with stroke) and 41 HCs were included in this study. Forty of the 48 individuals with TIA had motor impairment; specifically, 31 had impaired limb dexterity, four had limb weakness, and five had limb numbness.

### 2.2. Physiological and Biochemical Tests

Physiological and biochemical tests were completed for all participants within 24 hours prior to MRI scanning, including systolic blood pressure, diastolic blood pressure, blood sugar level, triglycerides, total cholesterol, high-density lipoprotein cholesterol (HDL-C), and low-density lipoprotein cholesterol (LDL-C). Based on age, blood pressure, clinical characteristics, symptom duration, and history of diabetes for each individual with TIA, we computed the ABCD2 score to assess the risk of subsequent stroke [5].

### 2.3. Data Acquisition

MRI scans were performed using a GE MR-750 3.0 T scanner (GE Medical Systems Inc., Waukesha, WI, United States). For individuals with TIA, there was a time interval from the latest TIA to the subsequent MRI scan of 6 hours to 16 days. All participants were instructed to relax, stay still, keep their eyes closed but not fall asleep, and not to think systematically during resting-state scanning [15]. Functional MRI scans were acquired using an echo planar imaging sequence with repetition time (TR)/echo time (TE) = 2000 ms/30 ms, flip angle (FA) = 60°, acquisition matrix = 64 × 64, slice thickness = 3.2 mm, gap = 0 mm, and slices = 43. The scanning time for the functional MRI was 8 minutes (240 volumes). The structural MRI data were acquired using a high-resolution anatomic sagittal 3D T1 sequence (voxel size = 1 mm × 1 mm × 1 mm), TR/TE = 8100 ms/3.1 ms, 176 slice acquisition matrix = 256 × 256, slice thickness = 1 mm, and gap = 0 mm. The scanning time for the structural MRI was 5 minutes.

### 2.4. Data Preprocessing

The Resting-State fMRI Data Analysis Toolkit plus (RESTplus V1.24, http://restfmri.net/forum/restplus) [57] was used in MATLAB 2017b to preprocess rs-fMRI images. For the preprocessing, we removed the first 10 volumes to ensure that the fMRI signal reached a steady state. Then, the rest of the volumes were slice timing corrected to the reference slice to synchronize timing across slices [58] and a six-parameter rigid-body transformation was used for realignment and motion correction [58, 59]. The realigned images were spatially normalized to the standard stereotactic space, as defined by the Montreal Neurological Institute (MNI) using the new segment method. The images were then smoothed using a Gaussian kernel with a full width at a half-maximum of 6 mm, which could improve the signal to noise ratio [12, 18, 60]. To control for motion and physiological nuisance signals, the Friston-24 parameters [61], white matter signals, and cerebrospinal fluid signals were regressed out [62]. Head motion could be further controlled by regressing the Friston-24 parameters, which include six head motion parameters generated by Realign, six head motion parameters at the preceding time point, and their corresponding 12 squared values [63, 64]. Detrending was performed to reduce the undesirable drift caused by the instrument, head motion, and physiological pulse aliasing [65, 66]. Grounded in the previous studies, band-pass filtering was not performed in the current study because of the low model order of GCA [38, 67–69].

### 2.5. Blind Deconvolution Procedure

Resting-state hemodynamic response function retrieval and deconvolution (RS-HRF, https://www.nitrc.org/projects/rshrf) was used to deconvolve the HRF from rs-fMRI signals, which could reduce the interindividual and interregional HRF variability and further improve the accuracy of effective connectivity estimation [69, 70].

### 2.6. Granger Causality Analysis

Bivariate voxel-wise GCA with the bilateral M1 as the ROIs was implemented using RESTplus. The ROIs were centered at *x* = −12, *y* = −30, and *z* = 54 and *x* = 12, *y* = −30, and *z* = 54 in the MNI space, with a radius of 6 mm [71]. GCA can estimate the causal effects from ROIs (*x*) to every other voxel in the whole brain (*y*) and the causal effects from every other voxel in the whole brain to ROIs [72, 73]. If the prediction of the future values of *y* can be improved by combining past values of *x* and *y* instead of using only past values of *y*, then *x* is said to Granger-cause *y*. Similarly, *y* is said to Granger-cause *x* when the future values of *x* are better predicted by combining the past values of *y* and *x* compared to using only the past values of *x* [36, 38, 74]. We used the signed-path coefficient GCA to reflect the alterations in effective connectivity among brain regions in the current study, and the increased or decreased effectivity activity between two brain regions was indicated by positive or negative GCA coefficients [75, 76]. Finally, the coefficient-based GCA values were converted into normally distributed *z* scores [76].

### 2.7. Statistical Analysis

The differences in age and clinical characteristics between individuals with TIA and HCs were analyzed using a two-sample *t*-test, and the differences in gender were analyzed using a chi-squared test. Statistical Product and Service Solutions (SPSS 20.0) was used in all of the analyses. Statistically significant between-group differences were set at *p* < 0.05.

Two-sample *t*-tests were conducted to determine the differences in effective connectivity between individuals with TIA and HCs using RESTplus. Subsequently, the Gaussian random field theory was adopted to carry out multiple comparison correction in the statistical analysis results (voxel *p* < 0.05, cluster *p* < 0.05, and two-tailed).

To detect the relationship between alterations of effective connectivity and clinical characteristics in TIA, we conducted partial correlation analysis between GCA values extracted from each region showing group differences and clinical characteristics with age and gender as covariates, including HDL-C, LDL-C, ABCD2 scores, and the time interval from the latest TIA to subsequent MRI scanning. Subsequently, Bonferroni correction was applied to correction for multiple comparison [77–79] and the threshold for statistical significance was set to *p* < 0.0125 (0.05/4).

## 3. Results

### 3.1. Demographic and Clinical Characteristics

In the present study, there were no significant differences in age (*p* = 0.182), gender (*p* = 0.670), triglyceride level (*p* = 0.213), and HDL-C level (*p* = 0.306). However, the systolic blood pressure (*p* < 0.001), diastolic blood pressure (*p* = 0.007), blood sugar level (*p* = 0.001), total cholesterol level (*p* = 0.045), and LDL-C level (*p* = 0.004) were significantly higher in the TIA group than in the HC group. The detailed demographic and clinical characteristics of the individuals with TIA and HCs are shown in Table 1.

### 3.2. GCA Results

#### 3.2.1. GCA Results from the Left M1 to the Whole Brain

**Figure 1:**
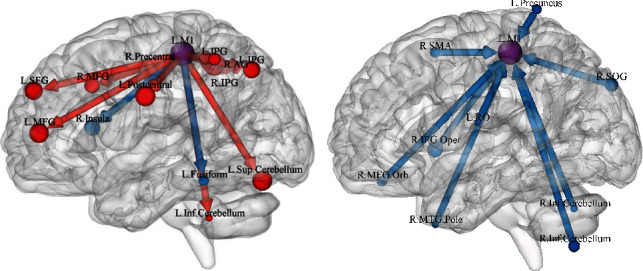
(a) Between-group differences in effective connectivity from the left M1 to the rest of the brain. (b) Between-group differences in effective connectivity from the rest of the brain to the left M1. The arrow indicated the causal inflow to the left M1 or the causal outflow from the left M1, and the red meant increased and blue meant decreased effective connectivity. L.M1: left primary motor cortex; R. Precentral: right precentral gyrus; R.IPG: right inferior parietal gyrus; L. Postcentral: left postcentral gyrus; L.Sup.Cerebellum: left superior cerebellum; L.MFG: left middle frontal gyrus; L.SFG: left superior frontal gyrus; L.IPG: left inferior parietal gyrus; R.MFG: right middle frontal gyrus; R.AG: right angular gyrus; L.Inf.Cerebellum: left inferior cerebellum; L. Fusiform: left fusiform; R. Insula: right insula; L.RO: left Rolandic operculum; R.MTG.Pole: temporal pole of the right middle temporal gyrus; R.Inf.Cerebellum: right inferior cerebellum; L. Precuneus: left precuneus; R.MFG.Orb: orbital part of the right middle frontal gyrus; R.SMA: right supplementary motor area; R.SOG: right superior occipital gyrus; R.IFG.Oper: opercular part of the right inferior frontal gyrus.

The GCA values in individuals with TIA from the left M1 to the right precentral gyrus, bilateral inferior parietal gyrus (IPG), left postcentral gyrus, left superior cerebellum, bilateral middle frontal gyrus (MFG), left superior frontal gyrus (SFG), right angular gyrus (AG), and left inferior cerebellum were higher, but the values in the left fusiform and right insula were lower than those in the HCs (Table 2, Figure 1(a)). Furthermore, brain maps of the alteration of effective connectivity from the left M1 to the whole brain are shown in the Supplementary Materials (Figure S1A).

#### 3.2.2. GCA Results from the Whole Brain to the Left M1

The GCA values in individuals with TIA from the left Rolandic operculum (RO), temporal pole of the right middle temporal gyrus (TP), right inferior cerebellum, left precuneus, orbital part of the right middle frontal gyrus, right supplementary motor area (SMA), right superior occipital gyrus (SOG), and opercular part of the right inferior frontal gyrus to the left M1 were lower than those in the HCs (Table 2, Figure 1(b)). Brain maps of the alteration of effective connectivity from the whole brain to the left M1 are shown in the Supplementary Materials (Figure S1B).

#### 3.2.3. GCA Results from the Right M1 to the Whole Brain

**Figure 2:**
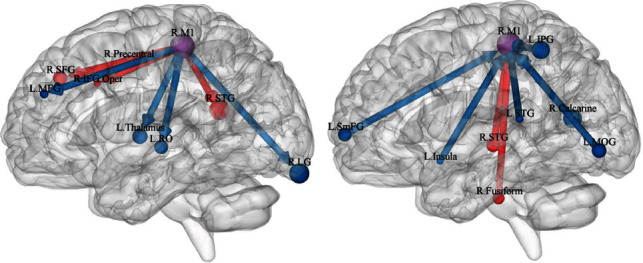
(a) Between-group differences in effective connectivity from the right M1 to the rest of the brain. (b) Between-group differences in effective connectivity from the rest of the brain to the right M1. The arrow indicated the causal inflow to the right M1 or the causal outflow from the right M1, and the red meant increased and blue meant decreased effective connectivity. R.STG: right superior temporal gyrus; R. Precentral: right precentral gyrus; R.SFG: right superior frontal gyrus; R.IFG.Oper: opercular part of the right inferior frontal gyrus; L.MFG: left middle frontal gyrus; L.RO: left Rolandic operculum; L.Thalamus: left thalamus; R.LG: right lingual gyrus; R. Fusiform: right fusiform; L. Insula: left insula; L.STG: left superior temporal gyrus; L.SmFG: left medial superior frontal gyrus; L.MOG: left middle occipital gyrus; L.IPG: left inferior parietal gyrus; R. Calcarine: right calcarine.

The GCA values in individuals with TIA from the right M1 to the right superior temporal gyrus (STG), right precentral gyrus, right SFG, and opercular part of the right inferior frontal gyrus were higher, but those in the left MFG, left RO, left thalamus, and right lingual gyrus (LG) were lower than those in the HCs (Table 3, Figure 2(a)). Brain maps of the alteration of effective connectivity from the right M1 to the whole brain are shown in Figure S2A of the Supplementary Materials.

#### 3.2.4. GCA Results from the Whole Brain to the Right M1

Compared with the HCs, the GCA values from the right STG and right fusiform to the right M1 were higher in individuals with TIA but those from the left insula, left medial SFG, left middle occipital gyrus (MOG), left IPG, and right calcarine were lower (Table 3, Figure 2(b)). Brain maps of the alteration of effective connectivity from the whole brain to the right M1 are shown in Figure S2B in the Supplementary Materials.

### 3.3. Partial Correlation between GCA and Clinical Characteristics

**Figure 3:**
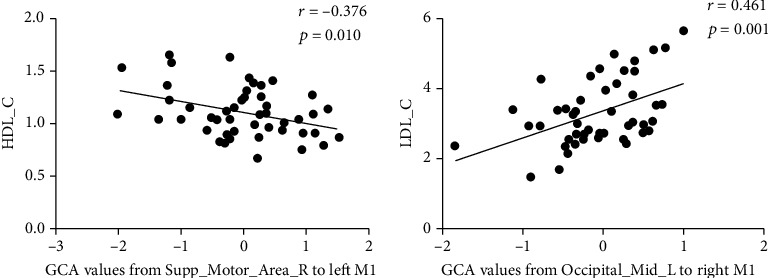
Partial correlation scatterplot between GCA values and the clinical characteristics. (a) Partial correlation scatterplot between GCA values from right SMA to left M1 and HDL-C. (b) Partial correlation scatterplot between GCA values from left MOG to right M1 and LDL-C.

Partial correlation analysis (covariates: age and gender) was conducted to investigate the relationship between the alterations of effective connectivity and clinical characteristics. After Bonferroni correction for multiple comparisons, we found that the GCA values from the right SMA to the left M1 were negatively associated with HDL-C (Figure 3(a)) but the GCA values from the left MOG to the right M1 were positively associated with LDL-C (Figure 3(b)). Specific information is provided in the Supplementary Materials (Tables S1–S4).

## 4. Discussion

In the present study, we used the GCA with the bilateral M1 as the ROIs to investigate the alteration of motor-related effective connectivity in individuals with TIA. Subsequently, we examined the correlation between altered effective connectivity and the clinical characteristics of individuals with TIA. Our results indicated that individuals with TIA had alterations in effective connectivity between the bilateral M1 and widely distributed brain regions, including motor control brain regions such as the SMA and cerebellum, as well as brain regions indirectly associated with motor function, such as the MOG, STG, and thalamus. These results have an important impact on understanding the pathological mechanisms of TIA from a new perspective and on reducing the risk of subsequent stroke.

The motor network mainly includes areas such as the M1, SMA, and cerebellum [80, 81], and the SMA is crucial for the initiation and control of the motor [82, 83]. Previous studies have demonstrated that stroke patients had decreased FC between the M1 and SMA [84], but it could be restored after treatment with repetitive transcranial magnetic stimulation (rTMS) using M1 as the target of stimulation [85]. Our results found decreased information inflow from the right SMA to the left M1 in TIA, which is consistent with previous studies and provides further directional information. This suggests that the poor motor performance of individuals with TIA may be related to impaired information transmission from the SMA to M1. Furthermore, we also found that decreased information inflow from the SMA to M1 was negatively correlated with HDL-C in individuals with TIA. Previous studies found that increased HDL-C was associated with a decreased risk of ischemic stroke [86, 87], which might suggest that lower effective connectivity from the right SMA to the left M1 is related to a higher risk of subsequent stroke. The cerebellum is involved in motor control as a balance center that coordinates the work of muscles [88–90]. Previous studies have shown that effective connectivity from M1 to the cerebellum was decreased whereas that from the cerebellum to M1 increased in stroke patients [34]. This is inconsistent with our results; we found increased information outflow from the left M1 to the left inferior cerebellum and left superior cerebellum, but the GCA values from the right inferior cerebellum to the left M1 demonstrated the opposite result. This might indicate that the motor impairment caused by TIA recovered and exhibited a bidirectional M1 and cerebellar neural circuit.

The precentral and postcentral gyri are also considered brain regions highly associated with motor function. The postcentral gyrus is one of the core nodes in the primary somatosensory cortex that can receive fiber information from spinal and bulbar motor neurons [91, 92]. The precentral gyrus, as part of the primary motor cortex, is primarily involved in motor execution [93, 94]. The current study showed that effective connectivity from the bilateral M1 to the right precentral gyrus and from the left M1 to the left postcentral gyrus was increased in individuals with TIA. This is consistent with previous FC studies, which found increased FC between the M1 and precentral gyrus in stroke patients and suggested that increased FC might be a compensatory strategy in the early poststroke period and gradually returned to normal levels after treatment [95]. Another study also found that rTMS treatment reduced FC between M1 and the ipsilateral postcentral gyrus [85]. Our results further provide directionality of the interactions between brain regions; that is, the increased FC between M1 and the precentral gyrus and postcentral gyrus might be related to the abnormal excess information transferred from M1 to these brain regions.

Vision and auditory can indirectly affect motor [96–98]. The temporal lobe is primarily responsible for auditory-motor processing skills [99]. Previous studies have demonstrated an increased information inflow from the ipsilateral M1 to the ipsilateral temporal lobe in stroke and have suggested that it might be associated with functional compensation [34]. Our results similarly found increased information outflow from the right M1 to the right STG in individuals with TIA and decreased information inflow from the right TP to the left M1. The increased information transfer between the ipsilateral M1 and temporal lobe may compensate for the information flow between the contralateral M1 and temporal lobe. The occipital lobe, LG, calcarine, fusiform, RO, and precuneus are all involved in the processing of visuospatial information, spatial attention, and vision-motion coordination [100–102]. Previous studies have demonstrated that stroke patients had decreased FC between the M1 and LG, calcarine, MOG, and precuneus [95, 103, 104]. This is consistent with our study, in which we found decreased information inflow from the left precuneus, right occipital lobe, and left RO to the left M1 and increased information inflow from the left occipital lobe and right calcarine to the right M1. This suggests that motor impairment in individuals with TIA might be related to reduced information transfer from these brain regions to the M1. It is noteworthy that the fusiform exhibited information flow in the opposite direction. Specifically, the effective connectivity from the left M1 to the left fusiform decreased but the effective connectivity from the right fusiform to the right M1 increased in the TIA. This suggests a possible functional compensation between the fusiform and M1. Our results also showed that elevated LDL-C was correlated with decreased information transfer from the visuomotor area to the right M1 and elevated LDL-C increased the risk of ischemic stroke [105, 106]. This indicates that impaired effective connectivity between M1 and vision-motion brain regions might not only indirectly affect motor performance in TIA but also increase the subsequent stroke risk in individuals with TIA.

Brain regions including the AG, thalamus, and insula are responsible for the integration of sensory information [85, 107, 108]. Previous studies have shown that the AG can deal with differences between the intended action and the motor result [109]. The thalamus, as a relay station for sensorimotor activity, is associated with motor observation and coordination [110]. We found that information outflow from the right M1 to the left thalamus was reduced in TIA, suggesting that motor impairment in TIA may be related to impaired transmission pathways between the M1 and thalamus. In addition, our study also found increased information outflow from the left M1 to the right AG but decreased information outflow to the right insula. This suggests that although the thalamus, insula, and AG are all associated with sensory integration and neural circuits from M1 to the thalamus and insula are impaired, those from M1 to the AG may be functionally compensated [111].

There are some potential limitations to the present study. First, it was conducted at only one hospital and had a relatively small sample size, which affects the generalizability of our results. In the future, the effective connectivity of TIA should be further explored through large-sample and multisite studies to better reveal the pathological mechanisms of TIA. Second, MRI scans were not conducted in individuals with TIA during the follow-up period, which prevented us from tracking how the motor-related effective connectivity network changed in individuals with TIA. Future longitudinal studies could track the alteration of information transfer between motor-related brain regions in TIA. Finally, the current study found that motor-related effective connectivity was associated with HDL-C and LDL-C levels in individuals with TIA but there was a lack of motor-related clinical data in individuals with TIA. Future studies should incorporate motor-related clinical measures to further elucidate the clinical significance of altered effective connectivity in TIA.

## 5. Conclusion

The voxel-wise GCA was used to explore the alteration of the motor-related effective connectivity network in TIA and suggested that the altered information flow between the M1 and right SMA and left MOG might be correlated with the risk of subsequent stroke attack in TIA. This contributes to our comprehension of the underlying pathological mechanism of motor impairment in TIA and suggested that effective connectivity could be beneficial for early screening and urgent intervention for TIA in the future.

## Figures and Tables

**Table 1:** Demographic and clinical characteristics for individuals with TIA and HCs.

	TIA group (*n* = 48)	HC group (*n* = 41)	*p* value
Age (years, mean ± SD)	57.604 ± 9.778	55.024 ± 8.033	0.182^a^
Gender (male/female)	37/11	30/11	0.670^b^
Systolic blood pressure (mmHg, mean ± SD)	145.542 ± 20.753	127.546 ± 19.527	<0.001^a^
Diastolic blood pressure (mmHg, mean ± SD)	86.667 ± 10.383	80.030 ± 10.896	0.007^a^
Blood sugar level (mmol/L, mean ± SD)	6.299 ± 2.113	5.120 ± 0.740	0.001^a^
Total cholesterol (mmol/L, mean ± SD)	5.242 ± 1.135	4.753 ± 1.011	0.045^a^
Triglycerides (mmol/L, mean ± SD)	1.603 ± 0.940	1.917 ± 1.345	0.213^a^
HDL-C (mmol/L, mean ± SD)	1.111 ± 0.238	1.051 ± 0.290	0.306^a^
LDL-C (mmol/L, mean ± SD)	3.314 ± 0.974	2.691 ± 0.904	0.004^a^
Motor impairment, no. (%)	40 (83.3%)	—	—
Time interval (days, mean ± SD)	2.610 ± 2.981	—	—
ABCD2 scores (median)	4 (2–6)	—	—

HDL-C: high-density lipoprotein cholesterol; LDL-C: low-density lipoprotein cholesterol; time interval: time interval from the latest TIA to subsequent MRI scanning. ^a^Calculated by two-sample *t*-test. ^b^Calculated by chi-squared *t*-test; there were 6 missing data of blood sugar level, total cholesterol, triglycerides, HDL-C, and LDL-C and 8 missing data of blood systolic pressure and blood diastolic pressure in the HC group.

**Table 2:** The differences of GCA with left M1 as ROI between the TIA and HC groups.

	Anatomical label	BA	Number of voxels	Peak MNI coordinates (*x*, *y*, *z*)	*t* value
*F*_*x*⟶*y*_					
	Precentral_R	4	74	57, −12, 45	4.43
	Parietal_Inf_R	40	59	48, −48, 48	4.31
	Postcentral_L	43	47	−57, −9, 30	4.10
	Cerebellum_6_L	18	43	−15, −75, −18	4.07
	Frontal_Mid_L	10	57	−24, 51, 9	4.04
	Frontal_Sup_L	9	45	−9, 54, 33	3.90
	Parietal_Inf_L	7	106	−33, −69, 45	3.83
	Frontal_Mid_R	44	47	39, 21, 36	3.67
	Parietal_Inf_L	40	48	−51, −48, 51	3.44
	Angular_R	39	47	42, −60, 45	3.04
	Cerebellum_8_L	NA	38	−18, −45, −39	2.91
	Fusiform_L	37	42	−21, −42, −18	−3.21
	Insula_R	48	76	33, 21, 12	−3.73

*F*_*y*⟶*x*_					
	Rolandic_Oper_L	48	74	−39, −12, 15	−3.58
	Temporal_Pole_Mid_R	20	96	48, 12, −42	−3.59
	Cerebellum_8_R	NA	65	12, −66, −33	−3.62
	Precuneus_L	5	68	−9, −45, 78	−3.82
	Frontal_Mid_Orb_R	11	265	27, 42, −18	−3.83
	Supp_Motor_Area_R	6	138	6, 12, 54	−3.87
	Cerebellum_8_R	NA	74	15, −66, −54	−3.96
	Occipital_Sup_R	19	321	21, −87, 36	−4.10
	Frontal_Inf_Oper_R	48	63	63, 12, 0	−4.29

Precentral_R: right precentral gyrus; Parietal_Inf_R: right inferior parietal gyrus; Postcentral_L: left postcentral gyrus; Cerebellum_6_L: left superior cerebellum; Frontal_Mid_L: left middle frontal gyrus; Frontal_Sup_L: left superior frontal gyrus; Parietal_Inf_L: left inferior parietal gyrus; Frontal_Mid_R: right middle frontal gyrus; Angular_R: right angular gyrus; Cerebellum_8_L: left inferior cerebellum; Fusiform_L: left fusiform; Insula_R: right insula; Rolandic_Oper_L: left Rolandic operculum; Temporal_Pole_Mid_R: temporal pole of the right middle temporal gyrus; Cerebellum_8_R: right inferior cerebellum; Precuneus_L: left precuneus; Frontal_Mid_Orb_R: orbital part of the right middle frontal gyrus; Supp_Motor_Area_R: right supplementary motor area; Occipital_Sup_R: right superior occipital gyrus; Frontal_Inf_Oper_R: opercular part of the right inferior frontal gyrus; BA: Brodmann area; MNI: Montreal Neurological Institute; NA: not available.

**Table 3:** The differences of GCA with the right M1 as ROI between the TIA and HC groups.

	Anatomical label	BA	Number of voxels	Peak MNI coordinates (*x*, *y*, *z*)	*t* value
*F*_*x*⟶*y*_					
	Temporal_Sup_R	22	351	60, −51, 18	4.39
	Precentral_R	6	55	48, 0, 45	3.77
	Frontal_Sup_R	9	58	24, 39, 36	3.76
	Frontal_Inf_Oper_R	44	51	39, 18, 33	3.04
	Frontal_Mid_L	46	40	−39, 48, 27	−3.18
	Rolandic_Oper_L	20	86	−30, −18, −3	−3.73
	Thalamus_L	NA	64	−6, −6, 3	−3.85
	Lingual_R	18	55	24, −96, −18	−4.32

*F*_*y*⟶*x*_					
	Temporal_Sup_R	21	231	51, −21, −3	4.02
	Fusiform_R	20	76	42, −24, −33	3.88
	Insula_L	48	101	−36, 9, −12	−3.44
	Temporal_Sup_L	42	104	−60, −36, 12	−3.53
	Frontal_Sup_Medial_L	10	389	−3, 63, 3	−4.02
	Occipital_Mid_L	19	389	−48, −81, −6	−4.11
	Parietal_Inf_L	40	463	−36, −48, 51	−4.43
	Calcarine_R	17	411	6, −66, 12	−4.46

Temporal_Sup_R: right superior temporal gyrus; Precentral_R: right precentral gyrus; Frontal_Sup_R: right superior frontal gyrus; Frontal_Inf_Oper_R: opercular part of the right inferior frontal gyrus; Frontal_Mid_L: left middle frontal gyrus; Rolandic_Oper_L: left Rolandic operculum; Thalamus_L: left thalamus; Lingual_R: right lingual gyrus; Fusiform_R: right fusiform; Insula_L: left insula; Temporal_Sup_L: left superior temporal gyrus; Frontal_Sup_Medial_L: left medial superior frontal gyrus; Occipital_Mid_L: left middle occipital gyrus; Parietal_Inf_L: left inferior parietal gyrus; Calcarine_R: right calcarine; BA: Brodmann area; MNI: Montreal Neurological Institute; NA: not available.

## Data Availability

All the data supporting the conclusions of this article are available from the authors upon reasonable request.

## Supplementary Materials

Supplementary Materials Figure S1: the results of GCA with left M1 as ROI. (A) Between-group differences in effective connectivity from left M1 to the rest of the brain. (B) Between-group differences in effective connectivity from the rest of the brain to left M1. Figure S2: the results of GCA with right M1 as ROI. (A) Between-group differences in effective connectivity from right M1 to the rest of the brain. (B) Between-group differences in effective connectivity from the rest of the brain to right M1. Table S1: the partial correlation between GCA values from left M1 to whole brain and the time interval from the latest TIA to subsequent MRI scanning in individuals with TIA. Table S2: the partial correlation between GCA values from whole brain to left M1 and the time interval from the latest TIA to subsequent MRI scanning in individuals with TIA. Table S3: the partial correlation between GCA values from right M1 to whole brain and the time interval from the latest TIA to subsequent MRI scanning in individuals with TIA. Table S4: the partial correlation between GCA values from whole brain to right M1 and the time interval from the latest TIA to subsequent MRI scanning in individuals with TIA.

## References

[1] Easton J. D., Saver J. L., Albers G. W. (2009). Definition and evaluation of transient ischemic attack a scientific statement for healthcare professionals from the American Heart Association/American Stroke Association Stroke Council; Council on Cardiovascular Surgery and Anesthesia; Council on Cardiovascular Radiology and Intervention; Council on Cardiovascular Nursing; and the Interdisciplinary Council on Peripheral Vascular Disease The American Academy of Neurology affirms the value of this statement as an educational tool for neurologists. *Stroke*.

[2] Feigin V., Norrving B., Sudlow C. L. M., Sacco R. L. (2018). Updated criteria for population-based stroke and transient ischemic attack incidence studies for the 21st century. *Stroke*.

[3] Lioutas V.-A., Ivan C. S., Himali J. J. (2021). Incidence of transient ischemic attack and association with long-term risk of stroke. *Jama-Journal of the American Medical Association*.

[4] Coull A. J., Lovett J. K., Rothwell P. M., Oxford Vascular Study (2004). Population based study of early risk of stroke after transient ischaemic attack or minor stroke: implications for public education and organisation of services. *BMJ (Clinical Research Ed.)*.

[5] Johnston S. C., Rothwell P. M., Nguyen-Huynh M. N. (2007). Validation and refinement of scores to predict very early stroke risk after transient ischaemic attack. *Lancet*.

[6] Amarenco P. (2020). Transient ischemic attack. *New England Journal of Medicine*.

[7] Lavallee P. C., Meseguer E., Abboud H. (2007). A transient ischaemic attack clinic with round-the-clock access (SOS-TIA): feasibility and effects. *The Lancet Neurology*.

[8] Rothwell P. M., Giles M. F., Chandratheva A. (2007). Effect of urgent treatment of transient ischaemic attack and minor stroke on early recurrent stroke (EXPRESS study): a prospective population-based sequential comparison. *Lancet (London, England)*.

[9] Gu H.-Q., Yang X., Wang C. J. (2021). Clinical characteristics, management, and in-hospital outcomes in patients with stroke or transient ischemic attack in China. *JAMA Network Open*.

[10] Shahjouei S., Sadighi A., Chaudhary D. (2021). A 5-decade analysis of incidence trends of ischemic stroke after transient ischemic attack a systematic review and meta-analysis. *JAMA Neurology*.

[11] Lv Y., Wei W., Han X. (2021). Multiparametric and multilevel characterization of morphological alterations in patients with transient ischemic attack. *Human Brain Mapping*.

[12] Lv Y., Wei W., Song Y. (2019). Non-invasive evaluation of cerebral perfusion in patients with transient ischemic attack: an fMRI study. *Journal of Neurology*.

[13] Su W., Guo J., Zhang Y. (2018). A longitudinal functional magnetic resonance imaging study of working memory in patients following a transient ischemic attack: a preliminary study. *Neuroscience Bulletin*.

[14] Lv Y., Han X., Song Y. (2019). Toward neuroimaging-based network biomarkers for transient ischemic attack. *Human Brain Mapping*.

[15] Biswal B., Zerrin Yetkin F., Haughton V. M., Hyde J. S. (1995). Functional connectivity in the motor cortex of resting human brain using echo- planar mri. *Magnetic Resonance in Medicine*.

[16] Fox M. D., Raichle M. E. (2007). Spontaneous fluctuations in brain activity observed with functional magnetic resonance imaging. *Nature Reviews. Neuroscience*.

[17] Ovadia-Caro S., Margulies D. S., Villringer A. (2014). The value of resting-state functional magnetic resonance imaging in stroke. *Stroke*.

[18] Lv Y., Li L., Song Y. (2019). The local brain abnormalities in patients with transient ischemic attack: a resting-state fMRI study. *Frontiers in Neuroscience*.

[19] Ma H., Huang G., Li M. (2021). The predictive value of dynamic intrinsic local metrics in transient ischemic attack. *Frontiers in Aging Neuroscience*.

[20] Guo J., Chen N., Li R. (2014). Regional homogeneity abnormalities in patients with transient ischaemic attack: a resting-state fMRI study. *Clinical Neurophysiology*.

[21] Gui S., Wang J., Wan H., Zhang J., Yang C. (2018). Altered intrinsic brain activities in patients with transient ischemic attack using amplitude of low-frequency fluctuation: a resting-state fMRI study. *International Journal of Clinical and Experimental Medicine*.

[22] Li R., Wang S., Zhu L. (2013). Aberrant functional connectivity of resting state networks in transient ischemic attack. *PLoS One*.

[23] Nicolas K., Goodin P., Visser M. M. (2021). Altered functional connectivity and cognition persists 4 years after a transient ischemic attack or minor stroke. *Frontiers in Neurology*.

[24] Zhu W., Che Y., Wang Y. (2019). Study on neuropathological mechanisms of primary monosymptomatic nocturnal enuresis in children using cerebral resting-state functional magnetic resonance imaging. *Scientific Reports*.

[25] Allegra M., Favaretto C., Metcalf N., Corbetta M., Brovelli A. (2021). Stroke-related alterations in inter-areal communication. *Neuroimage-Clinical*.

[26] Shi Y., Liu W., Liu R. (2019). Investigation of the emotional network in depression after stroke: a study of multivariate Granger causality analysis of fMRI data. *Journal of Affective Disorders*.

[27] Zhao Z., Cai H., Huang M. (2021). Altered functional connectivity of hippocampal subfields in poststroke dementia. *Journal of Magnetic Resonance Imaging*.

[28] Rolls E. T., Zhou Y., Cheng W., Gilson M., Deco G., Feng J. (2020). Effective connectivity in autism. *Autism Research*.

[29] Wang M., Zeng N., Zheng H., du X., Potenza M. N., Dong G. H. (2022). Altered effective connectivity from the pregenual anterior cingulate cortex to the laterobasal amygdala mediates the relationship between internet gaming disorder and loneliness. *Psychological Medicine*.

[30] Huang X., Zhang D., Wang P. (2021). Altered amygdala effective connectivity in migraine without aura: evidence from resting-state fMRI with Granger causality analysis. *The Journal of Headache and Pain*.

[31] Friston K. J. (1994). Functional and effective connectivity in neuroimaging: a synthesis. *Human Brain Mapping*.

[32] Li Z., Hu J., Wang Z., You R., Cao D. (2022). Basal ganglia stroke is associated with altered functional connectivity of the left inferior temporal gyrus. *Journal of Neuroimaging*.

[33] Liu F., Chen C. C., Hong W. J. (2022). Selectively disrupted sensorimotor circuits in chronic stroke with hand dysfunction. *CNS Neuroscience & Therapeutics*.

[34] Zhao Z., Wang X., Fan M. (2016). Altered effective connectivity of the primary motor cortex in stroke: a resting-state fMRI study with Granger causality analysis. *PLoS One*.

[35] Goebel R., Roebroeck A., Kim D. S., Formisano E. (2003). Investigating directed cortical interactions in time-resolved fMRI data using vector autoregressive modeling and Granger causality mapping. *Magnetic Resonance Imaging*.

[36] Roebroeck A., Formisano E., Goebel R. (2005). Mapping directed influence over the brain using Granger causality and fMRI. *NeuroImage*.

[37] Wang L., Cai J., Zhang M. (2016). Positive expression of human Cytomegalovirus phosphoprotein 65 in atherosclerosis. *BioMed Research International*.

[38] Hao Z., Shi Y., Huang L. (2022). The atypical effective connectivity of right temporoparietal junction in autism spectrum disorder: a multi-site study. *Frontiers in Neuroscience*.

[39] Feng Z., Xu S., Huang M., Shi Y., Xiong B., Yang H. (2016). Disrupted causal connectivity anchored on the anterior cingulate cortex in first-episode medication-naive major depressive disorder. *Progress in Neuro-Psychopharmacology & Biological Psychiatry*.

[40] Chand G. B., Dhamala M. (2017). Interactions between the anterior cingulate-insula network and the fronto- parietal network during perceptual decision-making. *NeuroImage*.

[41] Mozaffarian D., Benjamin E. J., Go A. S. (2015). Heart disease and stroke statistics--2015 update: a report from the American Heart Association. *Circulation*.

[42] Simmatis L., Krett J., Scott S. H., Jin A. Y. (2017). Robotic exoskeleton assessment of transient ischemic attack. *PLoS One*.

[43] Tsao C. W., Aday A. W., Almarzooq Z. I. (2022). Heart disease and stroke statistics-2022 update: a report from the American Heart Association. *Circulation*.

[44] Spark J. I., Blest N., Sandison S., Puckridge P. J., Saleem H. A., Russell D. A. (2011). Stroke and transient ischaemic attack awareness. *Medical Journal of Australia*.

[45] Low E., Crewther S. G., Ong B., Perre D., Wijeratne T. (2017). Compromised motor dexterity confounds processing speed task outcomes in stroke patients. *Frontiers in Neurology*.

[46] Lodha N., Patel P., Harrell J. (2019). Motor impairments in transient ischemic attack increase the odds of a positive diffusion-weighted imaging: a meta-analysis. *Restorative Neurology and Neuroscience*.

[47] Gladstone D. J., Kapral M. K., Fang J., Laupacis A., Tu J. V. (2004). Management and outcomes of transient ischemic attacks in Ontario. *CMAJ*.

[48] Lodha N., Harrell J., Eisenschenk S., Christou E. A. (2017). Motor impairments in transient ischemic attack increase the odds of a subsequent stroke: a meta-analysis. *Frontiers in Neurology*.

[49] Hatsopoulos N. G., Suminski A. J. (2011). Sensing with the motor cortex. *Neuron*.

[50] Naito E. (2004). Sensing limb movements in the motor cortex: how humans sense limb movement. *The Neuroscientist : A Review Journal Bringing Neurobiology, Neurology and Psychiatry*.

[51] Bajaj S., Butler A. J., Drake D., Dhamala M. (2015). Functional organization and restoration of the brain motor-execution network after stroke and rehabilitation. *Frontiers in Human Neuroscience*.

[52] Morrow M. M., Miller L. E. (2003). Prediction of muscle activity by populations of sequentially recorded primary motor cortex neurons. *Journal of Neurophysiology*.

[53] Gallego J. A., Perich M. G., Chowdhury R. H., Solla S. A., Miller L. E. (2020). Long-term stability of cortical population dynamics underlying consistent behavior. *Nature Neuroscience*.

[54] Figlewski K., Andersen H., Stærmose T., von Weitzel-Mudersbach P., Nielsen J. F., Blicher J. U. (2018). Decreased GABA levels in the symptomatic hemisphere in patients with transient ischemic attack. *Heliyon*.

[55] Radlinska B. A., Blunk Y., Leppert I. R., Minuk J., Pike G. B., Thiel A. (2012). Changes in callosal motor fiber integrity after subcortical stroke of the pyramidal tract. *Journal of Cerebral Blood Flow and Metabolism*.

[56] Nucera A., Azarpazhooh M. R., Cardinali L. (2017). Inhibition of the primary motor cortex and the upgoing thumb sign. *eNeurologicalSci*.

[57] Jia X. Z., Wang J., Sun H. Y. (2019). RESTplus: an improved toolkit for resting-state functional magnetic resonance imaging data processing. *Science Bulletin*.

[58] Chao-Gan Y., Yu-Feng Z. (2010). DPARSF: a MATLAB toolbox for “pipeline” data analysis of resting-state fMRI. *Frontiers in Systems Neuroscience*.

[59] Power J. D., Schlaggar B. L., Petersen S. E. (2015). Recent progress and outstanding issues in motion correction in resting state fMRI. *NeuroImage*.

[60] Petersson K. M., Nichols T. E., Poline J. B., Holmes A. P. (1999). Statistical limitations in functional neuroimaging. II. Signal detection and statistical inference. *Philosophical Transactions of the Royal Society of London. Series B, Biological Sciences*.

[61] Friston K. J., Williams S., Howard R., Frackowiak R. S. J., Turner R. (1996). Movement-related effects in fMRI time-series. *Magnetic Resonance in Medicine*.

[62] Fox M. D., Snyder A. Z., Vincent J. L., Corbetta M., van Essen D. C., Raichle M. E. (2005). The human brain is intrinsically organized into dynamic, anticorrelated functional networks. *Proceedings of the National Academy of Sciences of the United States of America*.

[63] Chen J. E., Glover G. H. (2015). Functional magnetic resonance imaging methods. *Neuropsychology Review*.

[64] Yan C.-G., Cheung B., Kelly C. (2013). A comprehensive assessment of regional variation in the impact of head micromovements on functional connectomics. *NeuroImage*.

[65] Turner R. (1997). Signal sources in bold contrast fMRI. *Advances in Experimental Medicine and Biology*.

[66] Lowe M. J., Russell D. P. (1999). Treatment of baseline drifts in fMRI time series analysis. *Journal of Computer Assisted Tomography*.

[67] Hamilton J. P., Chen G., Thomason M. E., Schwartz M. E., Gotlib I. H. (2011). Investigating neural primacy in major depressive disorder: multivariate Granger causality analysis of resting-state fMRI time-series data. *Molecular Psychiatry*.

[68] Liao W., Ding J., Marinazzo D. (2011). Small-world directed networks in the human brain: multivariate Granger causality analysis of resting-state fMRI. *NeuroImage*.

[69] Wu G. R., Liao W., Stramaglia S., Ding J. R., Chen H., Marinazzo D. (2013). A blind deconvolution approach to recover effective connectivity brain networks from resting state fMRI data. *Medical Image Analysis*.

[70] Wu G. R., Colenbier N., van den Bossche S. (2021). rsHRF: a toolbox for resting-state HRF estimation and deconvolution. *NeuroImage*.

[71] Liu J., Qin W., Zhang J., Zhang X., Yu C. (2015). Enhanced interhemispheric functional connectivity compensates for anatomical connection damages in subcortical stroke. *Stroke*.

[72] Wang T., Chen N., Zhan W. (2016). Altered effective connectivity of posterior thalamus in migraine with cutaneous allodynia: a resting-state fMRI study with granger causality analysis. *Journal of Headache and Pain*.

[73] Wei H.-L., Chen J., Chen Y. C. (2020). Impaired effective functional connectivity of the sensorimotor network in interictal episodic migraineurs without aura. *Journal of Headache and Pain*.

[74] Granger C. W. J. (1969). Investigating causal relations by econometric models and cross-spectral methods. *Econometrica*.

[75] Chen G., Hamilton J. P., Thomason M. E., Gotlib I. H., Saad Z. S., Cox R. W. (2009). Granger causality via vector autoregression tuned for FMRI data. *Analysis*.

[76] Zang Z. X., Yan C. G., Dong Z. Y., Huang J., Zang Y. F. (2012). Granger causality analysis implementation on MATLAB: a graphic user interface toolkit for fMRI data processing. *Journal of Neuroscience Methods*.

[77] Nugent A. C., Martinez A., D'Alfonso A., Zarate C. A., Theodore W. H. (2015). The relationship between glucose metabolism, resting-state fMRI BOLD signal, and GABAA-binding potential: a preliminary study in healthy subjects and those with temporal lobe epilepsy. *Journal of Cerebral Blood Flow and Metabolism*.

[78] Higgins J. P., Elliott J. M., Parrish T. B. (2020). Brain network disruption in whiplash. *American Journal of Neuroradiology*.

[79] Liu Y., Chen Y., Liang X. (2020). Altered resting-state functional connectivity of multiple networks and disrupted correlation with executive function in major depressive disorder. *Frontiers in Neurology*.

[80] Middleton F. A., Strick P. L. (2000). Basal ganglia and cerebellar loops: motor and cognitive circuits. *Brain Research. Brain Research Reviews*.

[81] Tang Q., Li G., Liu T. (2015). Modulation of interhemispheric activation balance in motor-related areas of stroke patients with motor recovery: systematic review and meta-analysis of fMRI studies. *Neuroscience and Biobehavioral Reviews*.

[82] Nutt J. G., Bloem B. R., Giladi N., Hallett M., Horak F. B., Nieuwboer A. (2011). Freezing of gait: moving forward on a mysterious clinical phenomenon. *Lancet Neurology*.

[83] Lindenbach D., Bishop C. (2013). Critical involvement of the motor cortex in the pathophysiology and treatment of Parkinson’s disease. *Neuroscience and Biobehavioral Reviews*.

[84] Zheng X., Sun L., Yin D. (2016). The plasticity of intrinsic functional connectivity patterns associated with rehabilitation intervention in chronic stroke patients. *Neuroradiology*.

[85] Li J., Zhang X. W., Zuo Z. T. (2016). Cerebral functional reorganization in ischemic stroke after repetitive transcranial magnetic stimulation: an fMRI study. *CNS Neuroscience & Therapeutics*.

[86] Amarenco P., Labreuche J., Touboul P.-J. (2008). High-density lipoprotein-cholesterol and risk of stroke and carotid atherosclerosis: a systematic review. *Atherosclerosis*.

[87] Qie R., Liu L., Zhang D. (2021). Dose-response association between high-density lipoprotein cholesterol and stroke: a systematic review and meta-analysis of prospective cohort studies. *Preventing Chronic Disease*.

[88] Xie Z., Cui F., Zou Y., Bai L. (2014). Acupuncture enhances effective connectivity between cerebellum and primary sensorimotor cortex in patients with stable recovery stroke. *Evidence-based Complementary and Alternative Medicine*.

[89] Bastian A. J. (2011). Moving, sensing and learning with cerebellar damage. *Current Opinion in Neurobiology*.

[90] Müller M. L. T. M., Albin R. L., Kotagal V. (2013). Thalamic cholinergic innervation and postural sensory integration function in Parkinson’s disease. *Brain*.

[91] Li X. G., Florence S. L., Kaas J. H. (1990). Areal distributions of cortical neurons projecting to different levels of the caudal brain stem and spinal cord in rats. *Somatosensory & Motor Research*.

[92] Haseeb A., Asano E., Juhász C., Shah A., Sood S., Chugani H. T. (2007). Young patients with focal seizures may have the primary motor area for the hand in the postcentral gyrus. *Epilepsy Research*.

[93] Gao J., Yang C., Li Q. (2021). Hemispheric difference of regional brain function exists in patients with acute stroke in different cerebral hemispheres: a resting-state fMRI study. *Neuroscience*.

[94] Sandman C. A., Buss C., Head K., Davis E. P. (2015). Fetal exposure to maternal depressive symptoms is associated with cortical thickness in late childhood. *Biological Psychiatry*.

[95] Wang H., Xu G., Wang X. (2019). The reorganization of resting-state brain networks associated with motor imagery training in chronic stroke patients. *IEEE Transactions on Neural Systems and Rehabilitation Engineering*.

[96] Secoli R., Milot M. H., Rosati G., Reinkensmeyer D. J. (2011). Effect of visual distraction and auditory feedback on patient effort during robot-assisted movement training after stroke. *Journal of Neuroengineering and Rehabilitation*.

[97] Berger C. C., Ehrsson H. H. (2018). Mental imagery induces cross-modal sensory plasticity and changes future auditory perception. *Psychological Science*.

[98] Reh J., Schmitz G., Hwang T. H., Effenberg A. O. (2022). Loudness affects motion: asymmetric volume of auditory feedback results in asymmetric gait in healthy young adults. *BMC Musculoskeletal Disorders*.

[99] Tsai C. G., Fan L. Y., Lee S. H., Chen J. H., Chou T. L. (2012). Specialization of the posterior temporal lobes for audio-motor processing – evidence from a functional magnetic resonance imaging study of skilled drummers. *The European Journal of Neuroscience*.

[100] Margulies D. S., Vincent J. L., Kelly C. (2009). Precuneus shares intrinsic functional architecture in humans and monkeys. *Proceedings of the National Academy of Sciences of the United States of America*.

[101] Liu H., Chen L., Zhang G. (2020). Scalp acupuncture enhances the functional connectivity of visual and cognitive-motor function network of patients with acute ischemic stroke. *Evidence-based Complementary and Alternative Medicine*.

[102] Deconinck F. J. A., Smorenburg A. R. P., Benham A., Ledebt A., Feltham M. G., Savelsbergh G. J. P. (2015). Reflections on mirror therapy: a systematic review of the effect of mirror visual feedback on the brain. *Neurorehabilitation and Neural Repair*.

[103] Li J., Cheng L., Chen S. (2022). Functional connectivity changes in multiple-frequency bands in acute basal ganglia ischemic stroke patients: a machine learning approach. *Neural Plasticity*.

[104] Chen J., Sun D., Shi Y. (2018). Alterations of static functional connectivity and dynamic functional connectivity in motor execution regions after stroke. *Neuroscience Letters*.

[105] Sun L., Clarke R., Bennett D. (2019). Causal associations of blood lipids with risk of ischemic stroke and intracerebral hemorrhage in Chinese adults. *Nature Medicine*.

[106] Pan Y., Wangqin R., Li H. (2022). LDL-C levels, lipid-lowering treatment and recurrent stroke in minor ischaemic stroke or TIA. *Stroke and Vascular Neurology*.

[107] Seghier M. L. (2013). The angular gyrus: multiple functions and multiple subdivisions. *The Neuroscientist*.

[108] Farrer C., Frey S. H., van Horn J. D. (2008). The angular gyrus computes action awareness representations. *Cerebral Cortex*.

[109] Gandolla M., Niero L., Molteni F., Guanziroli E., Ward N. S., Pedrocchi A. (2021). Brain plasticity mechanisms underlying motor control reorganization: pilot longitudinal study on post-stroke subjects. *Brain Sciences*.

[110] Brunner I. C., Skouen J. S., Ersland L., Grüner R. (2014). Plasticity and response to action observation: a longitudinal fMRI study of potential mirror neurons in patients with subacute stroke. *Neurorehabilitation and Neural Repair*.

[111] Zhang Y., Liu H., Wang L. (2016). Relationship between functional connectivity and motor function assessment in stroke patients with hemiplegia: a resting-state functional MRI study. *Neuroradiology*.

