# Supplementary material for: Altered Effective Connectivity of the Primary Motor Cortex in Transient Ischemic Attack
Source: Neural Plast. 2022 Nov 18;2022:2219993. doi: 10.1155/2022/2219993 (PMC9699783; doi:10.1155/2022/2219993)
Supplement: Supplementary Materials — Figure S1: the results of GCA with left M1 as ROI. (A) Between-group differences in effective connectivity from left M1 to the rest of the brain. (B) Between-group differences in effective connectivity from the rest of the brain to left M1. Figure S2: the results of GCA with right M1 as ROI. (A) Between-group differences in effective connectivity from right M1 to the rest of the brain. (B) Between-group differences in effective connectivity from the rest of the brain to right M1. Table S1: the partial correlation between GCA values from left M1 to whole brain and the time interval from the latest TIA to subsequent MRI scanning in individuals with TIA. Table S2: the partial correlation between GCA values from whole brain to left M1 and the time interval from the latest TIA to subsequent MRI scanning in individuals with TIA. Table S3: the partial correlation between GCA values from right M1 to whole brain and the time interval from the latest TIA to subsequent MRI scanning in individuals with TIA. Table S4: the partial correlation between GCA values from whole brain to right M1 and the time interval from the latest TIA to subsequent MRI scanning in individuals with TIA. [file 2219993.f1.docx]

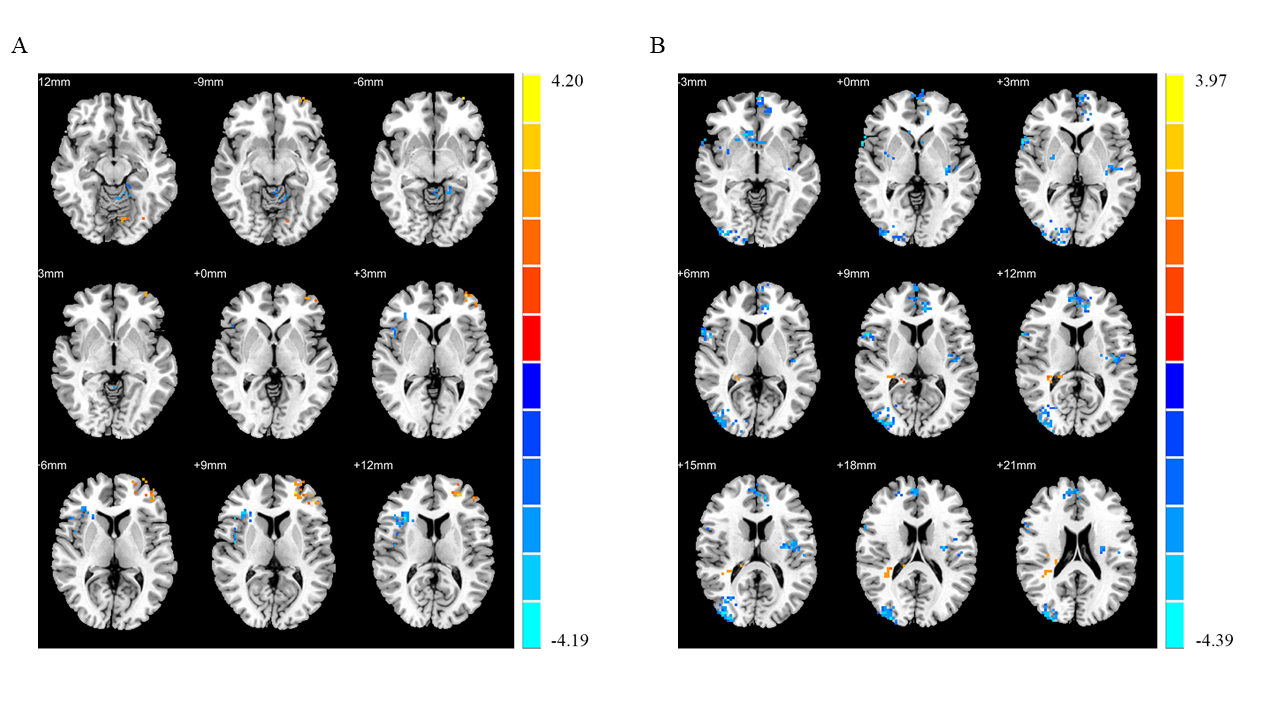


**Figure S1** The results of GCA with left M1 as ROI. (A) Between-group differences in effective connectivity from left M1 to the rest of the brain. (B) Between-group differences in effective connectivity from the rest of the brain to left M1.


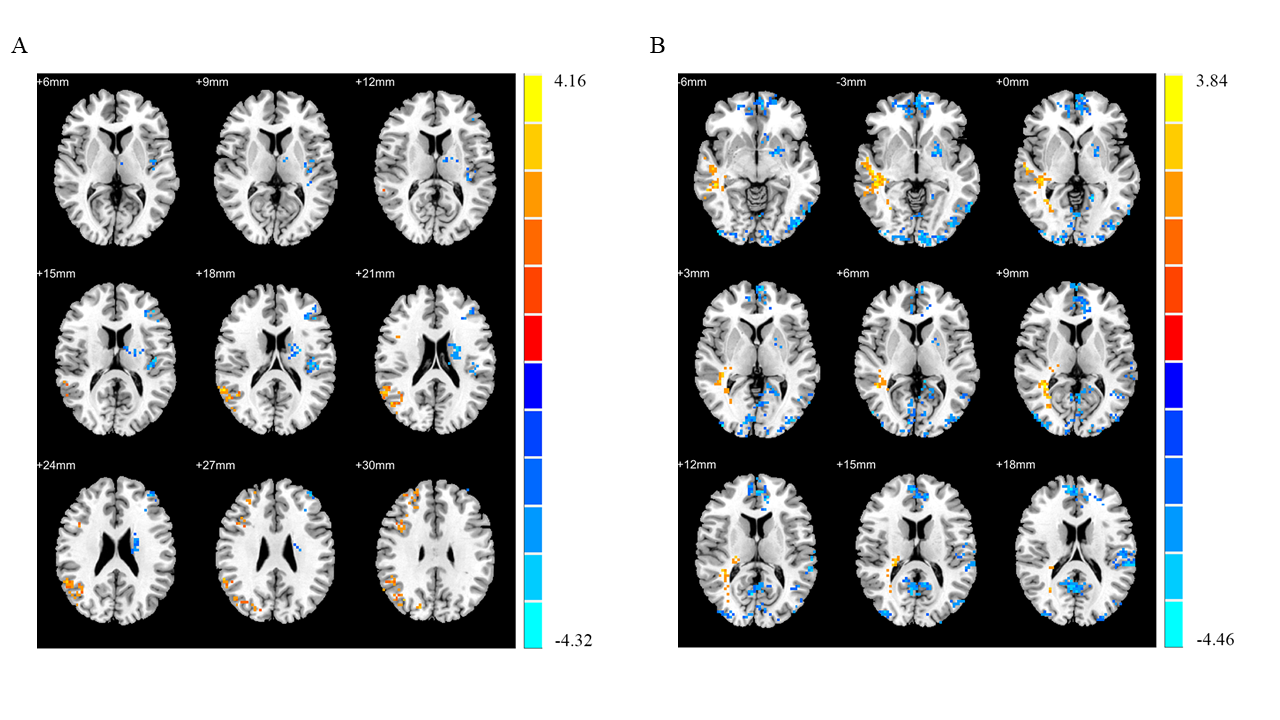


**Figure S2** The results of GCA with right M1 as ROI. (A) Between-group differences in effective connectivity from right M1 to the rest of the brain. (B) Between-group differences in effective connectivity from the rest of the brain to right M1.

**Table S1** The partial correlation between GCA values from left M1 to whole brain and the time interval from latest TIA to subsequent MRI scanning in individuals with TIA.

| GCA values | HDL-C | | LDL-C | | ABCD 2 | | time interval | |
| --- | --- | --- | --- | --- | --- | --- | --- | --- |
|  | *r* | *p* | *r* | *p* | *r* | *p* | *r* | *p* |
| Cerebelum_8_L | 0.327 | 0.027 | 0.057 | 0.707 | 0.191 | 0.204 | 0.168 | 0.265 |
| Cerebelum_6_L | 0.223 | 0.137 | 0.278 | 0.061 | -0.044 | 0.774 | 0.117 | 0.440 |
| Fusiform_L | 0.009 | 0.952 | 0.057 | 0.706 | 0.056 | 0.713 | -0.082 | 0.589 |
| Frontal_Mid_L | 0.266 | 0.074 | 0.130 | 0.388 | 0.040 | 0.793 | -0.094 | 0.535 |
| Insula_R | 0.167 | 0.267 | 0.105 | 0.487 | 0.271 | 0.068 | -0.210 | 0.161 |
| Precentral_R | 0.105 | 0.486 | 0.128 | 0.397 | 0.107 | 0.480 | -0.166 | 0.269 |
| Postcentral_L | 0.085 | 0.572 | 0.004 | 0.979 | 0.170 | 0.258 | 0.004 | 0.980 |
| Frontal_Sup_L | 0.191 | 0.204 | 0.105 | 0.486 | 0.146 | 0.334 | 0.067 | 0.656 |
| Frontal_Mid_R | 0.277 | 0.062 | 0.356 | 0.015 | 0.321 | 0.030 | -0.119 | 0.430 |
| Parietal_Inf_L | 0.355 | 0.016 | 0.304 | 0.040 | -0.160 | 0.288 | 0.131 | 0.384 |
| Angular_R | 0.149 | 0.322 | 0.234 | 0.118 | 0.158 | 0.295 | -0.256 | 0.085 |
| Parietal_Inf_L | 0.147 | 0.328 | 0.283 | 0.057 | -0.068 | 0.651 | 0.021 | 0.892 |
| Parietal_Inf_R | 0.104 | 0.491 | 0.324 | 0.028 | 0.063 | 0.678 | 0.071 | 0.638 |

Abbreviation: GCA, *Granger causality analysis*; HDL-C, *High-Density Lipoprotein Cholesterol*; LDL-C, *Low-Density Lipoprotein Cholesterol*. Time interval, *time interval between the latest TIA and subsequent MRI scanning*; r, *partial correlation with age and sex as covariates*; p, *significance*; ^*^*p* < 0.0125

**Table S2** The partial correlation between GCA values from whole brain to left M1 and the time interval from latest TIA to subsequent MRI scanning in individuals with TIA.

| GCA values | HDL-C | | LDL-C | | ABCD 2 | | time interval | | |
| --- | --- | --- | --- | --- | --- | --- | --- | --- | --- |
|  | *r* | *p* | *r* | *p* | *r* | *p* | *r* | *p* | |
| Cerebelum_8_R | -0.172 | 0.252 | -0.077 | 0.609 | -0.005 | 0.973 | -0.244 | | 0.102 |
| Temporal_Pole_Mid_R | 0.197 | 0.188 | 0.071 | 0.640 | 0.227 | 0.129 | -0.211 | | 0.140 |
| Cerebelum_8_R | -0.037 | 0.806 | 0.042 | 0.784 | 0.160 | 0.289 | -0.152 | | 0.313 |
| Frontal_Mid_Orb_R | 0.234 | 0.118 | 0.148 | 0.327 | 0.089 | 0.557 | 0.158 | | 0.294 |
| Occipital_Sup_R | 0.052 | 0.729 | -0.068 | 0.654 | 0.350 | 0.017 | -0.179 | | 0.233 |
| Rolandic_Oper_L | 0.073 | 0.628 | -0.062 | 0.682 | 0.075 | 0.621 | -0.148 | | 0.326 |
| Frontal_Inf_Oper_R | 0.158 | 0.294 | 0.259 | 0.082 | -0.043 | 0.777 | -0.147 | | 0.330 |
| Precuneus_L | -0.129 | 0.394 | 0.038 | 0.800 | -0.004 | 0.977 | -0.018 | | 0.903 |
| Supp_Motor_Area_R | -0.376 | 0.010^*^ | 0.055 | 0.717 | -0.207 | 0.167 | 0.069 | | 0.648 |

Abbreviation: GCA, *Granger causality analysis*; HDL-C, *High-Density Lipoprotein Cholesterol*; LDL-C, *Low-Density Lipoprotein Cholesterol*. Time interval, *time interval between the latest TIA and subsequent MRI scanning*; r, *partial correlation with age and sex as covariates*; p, *significance*; ^*^*p* < 0.0125

**Table S3** The partial correlation between GCA values from right M1 to whole brain and the time interval from latest TIA to subsequent MRI scanning in individuals with TIA.

| GCA values | HDL-C | | LDL-C | | ABCD 2 | | time interval | | |
| --- | --- | --- | --- | --- | --- | --- | --- | --- | --- |
|  | *r* | *p* | *r* | *p* | *r* | *p* | *r* | *p* | |
| Lingual_R | -0.129 | 0.394 | 0.097 | 0.519 | -0.102 | 0.500 | -0.088 | | 0.561 |
| Rolandic_Oper_L | 0.079 | 0.603 | -0.190 | 0.205 | 0.323 | 0.029 | -0.135 | | 0.372 |
| Thalamus_L | -0.018 | 0.907 | -0.085 | 0.573 | 0.083 | 0.585 | -0.218 | | 0.145 |
| Frontal_Mid_L | 0.125 | 0.408 | -0.047 | 0.757 | -0.100 | 0.510 | 0.131 | | 0.386 |
| Temporal_Sup_R | -0.032 | 0.835 | 0.007 | 0.962 | -0.033 | 0.826 | 0.028 | | 0.852 |
| Frontal_Inf_Oper_R | 0.114 | 0.452 | 0.015 | 0.924 | 0.019 | 0.899 | -0.132 | | 0.381 |
| Frontal_Sup_R | 0.151 | 0.315 | 0.041 | 0.785 | 0.105 | 0.486 | -0.032 | | 0.831 |
| Precentral_R | 0.283 | 0.057 | -0.066 | 0.663 | 0.127 | 0.401 | -0.181 | | 0.229 |

Abbreviation: GCA, *Granger causality analysis*; HDL-C, *High-Density Lipoprotein Cholesterol*; LDL-C, *Low-Density Lipoprotein Cholesterol*. Time interval, *time interval between the latest TIA and subsequent MRI scanning*; r, *partial correlation with age and sex as covariates*; p, *significance*; ^*^*p* < 0.0125

**Table S4** The partial correlation between GCA values from whole brain to right M1 and the time interval from latest TIA to subsequent MRI scanning in individuals with TIA.

| GCA values | HDL-C | | LDL-C | | ABCD 2 | | time interval | |
| --- | --- | --- | --- | --- | --- | --- | --- | --- |
|  | *r* | *p* | *r* | *p* | *r* | *p* | *r* | *p* |
| Fusiform_R | -0.052 | 0.732 | -0.142 | 0.348 | -0.068 | 0.652 | 0.266 | 0.074 |
| Occipital_Mid_L | 0.205 | 0.173 | 0.461 | 0.001^*^ | -0.106 | 0.483 | -0.172 | 0.254 |
| Temporal_Sup_R | -0.030 | 0.841 | -0.002 | 0.989 | -0.157 | 0.296 | 0.230 | 0.125 |
| Frontal_Sup_Medial_L | 0.317 | 0.032 | 0.048 | 0.751 | 0.073 | 0.632 | 0.026 | 0.866 |
| Calcarine_R | 0.125 | 0.410 | 0.278 | 0.061 | -0.072 | 0.635 | 0.070 | 0.643 |
| Insula_L | 0.316 | 0.033 | 0.085 | 0.574 | 0.031 | 0.838 | -0.165 | 0.274 |
| Temporal_Sup_L | 0.161 | 0.287 | -0.063 | 0.679 | 0.047 | 0.756 | -0.006 | 0.966 |
| Parietal_Inf_L | -0.088 | 0.560 | 0.260 | 0.082 | -0.307 | 0.038 | 0.005 | 0.976 |

Abbreviation: GCA, *Granger causality analysis*; HDL-C, *High-Density Lipoprotein Cholesterol*; LDL-C, *Low-Density Lipoprotein Cholesterol*. Time interval, *time interval between the latest TIA and subsequent MRI scanning*; r, *partial correlation with age and sex as covariates*; p, *significance*; ^*^*p* < 0.0125
